# The role of guilt in Posttraumatic Stress Disorder

**DOI:** 10.1080/20008198.2017.1407202

**Published:** 2017-12-05

**Authors:** Konstantin Bub, Miriam J. J. Lommen

**Affiliations:** ^a^ Clinical Psychology and Experimental Psychopathology, University of Groningen, Groningen, The Netherlands

**Keywords:** Trauma, PTSD, intrusions, emotion, distress, guilt, • Short-term feelings of guilt following an analogue trauma or stressor in the laboratory can be induced in healthy participants. • Feelings of guilt as a reaction to a stressor were related to a higher number of stressor-related intrusions and higher associated distress. • Feelings of guilt may contribute to the development of PTSD symptoms such as intrusive thoughts. • This study supports the potential importance of attending to feelings of guilt in PTSD, besides feelings of anxiety.

## Abstract

**Background**: A growing body of evidence supports the notion that the emotional profile of Posttraumatic Stress Disorder (PTSD) may be more diverse than traditional accounts presume. PTSD’s image as an anxiety-based disorder is undergoing change as the significance of other emotions in its development becomes more evident. Experimental research is needed in order to expand the understanding of underlying processes driving the development of PTSD.

**Objective**: Experimentally test the influence of stressor-related guilt on the occurrence of PTSD symptomatology.

**Method**: A non-clinical student sample faced an analogue trauma, a stressor in the form of a computer crash and related loss of data. We either personally blamed participants for causing the incident (blame group) or told them that it was a technical failure and therefore not their fault (no-blame group). Levels of guilt before and after the incident as well as number and associated distress of incident-related intrusions were assessed using a one-day diary and compared between groups.

**Results**: The guilt manipulation was successful: feelings of guilt significantly increased in the blame group but not in the no-blame group. Furthermore, the blame group showed a significantly higher number of intrusions and associated distress compared to the no-blame group at one-day follow-up.

**Conclusions**: These laboratory findings indicate that feelings of guilt may lead to increased PTSD symptomatology, supporting the view that guilt experienced in reaction to a traumatic event may be part of a causal mechanism driving the development of PTSD.

## El papel de la culpa en el trastorno de estrés postraumático

**Planteamiento:** Un creciente conjunto de evidencias apoya la noción de que el perfil emocional del Trastorno por Estrés Postraumático (TEPT) puede ser más diverso de lo que suponen las consideraciones tradicionales. La imagen del TEPT como un trastorno basado en la ansiedad está experimentando cambios a medida que se hace más evidente la importancia de otras emociones en su desarrollo. Se necesita investigación experimental para ampliar la comprensión de los procesos subyacentes que fomentan el desarrollo del trastorno por estrés postraumático.

**Objetivo:** Probar experimentalmente la influencia de la culpa relacionada con el factor de estrés en la ocurrencia de la sintomatología del TEPT.

**Método:** Una muestra no clínica de estudiantes se enfrentó a un trauma análogo, que es un factor de estrés en forma de fallo informático y la consiguiente pérdida de datos. O bien culpamos personalmente a los participantes por causar el incidente (culpan al grupo) o les dijimos que se trataba de un fallo técnico y, por lo tanto, no era su culpa (no culpan al grupo). Se evaluaron los niveles de culpabilidad antes y después del incidente, así como la cantidad de intrusiones relacionadas con el incidente y la angustia asociada usando un diario de 1 día y se compararon entre ambos grupos.

**Resultados:** La manipulación de la culpa tuvo éxito, ya que los sentimientos de culpa aumentaron significativamente en el grupo al que se culpó, pero no en el grupo al que no se culpó. Además, el grupo de la culpa mostró un número significativamente mayor de intrusiones y de angustia asociada en comparación con el grupo sin culpa en el seguimiento 1 día después.

**Conclusiones:** Estos hallazgos de laboratorio indican que los sentimientos de culpa pueden conducir a una mayor sintomatología del TEPT, lo que respalda la opinión de que la culpa experimentada en reacción a un evento traumático puede ser parte de un mecanismo causal que fomenta el desarrollo del TEPT.

## 创伤后应激障碍中内疚的作用

**背景** ：支持创伤后应激障碍（PTSD）的情绪剖面的证据逐渐增多。PTSD 发展过程中的其它情绪变得越来越受关注，将PTSD看做是基于焦虑的失调这种观点已经正在改变。需要实验研究来增进对影响PTSD 发展的潜在过程的理解。

**目标** ：使用实验考察压力相关的内疚在PTSD症状发生中的影响。

**方法**：在一个非临床的学生样本中使用模拟创伤的方法，在假装发生电脑崩溃并丢失了数据后，我们人为地谴责被试引起了这个事故（谴责组），或者告诉他们这是技术故障因此不是他们的问题（非谴责组）。使用‘一天日记法’记录事故之前和之后的内疚水平和数量、事件相关的闯入，并进行了组间比较。

**结果**：内疚操作是成功的，因为内疚感在谴责组里显著增加，但非谴责组没有增加。同时，与非谴责组相比，谴责组在一天后表现出显著更多的闯入和相关的痛苦。

**结论**：这些实验室发现提示内疚感可能会导致PTSD症状增加，支持了讲内疚体验看做是对创伤事件的反应可能是导致PTSD发展的因果机制的一部分。

In recent years a process of rethinking has occurred in the research on Posttraumatic Stress Disorder (PTSD). A growing body of evidence now indicates that, contrary to traditional accounts, PTSD may not primarily be an anxiety-based disorder. Instead, a wide range of other emotions accompanies PTSD and may be central to its development and maintenance (Lee, Scragg, & Turner, 2001; Power & Fyvie, 2012). Accordingly, the Diagnostic and Statistical Manual of Mental Disorders (DSM-5; American Psychiatric Association, 2013) changed its classification of PTSD from anxiety-related disorder to trauma- and stressor-related disorder and added anger, guilt, and shame alongside fear as significant emotional responses to trauma.

The emotion of guilt in particular has been consistently linked to the development and maintenance of PTSD symptomatology (Kubany & Watson, 2003; Lee et al., 2001; Wilson, Drozdek, & Turkovic, 2006). Using a self-report measure, Henning and Frueh (1997) found severity of combat-related guilt in veterans to be positively correlated with re-experiencing and avoidance symptoms of PTSD, as well as with a general measure of PTSD severity. Another study by Beck et al. (2011) found guilt-related distress and cognitions to be positively associated with PTSD severity in a cross-sectional study with women experiencing intimate partner violence. Despite sound evidence for a guilt-PTSD link, the exact nature of the relationship remains evasive and in pressing need of further clarification. Pugh, Taylor, and Berry (2015) presented four putative models of the association between guilt and PTSD: (1) trauma-related guilt plays a causal role in the development of PTSD symptomatology; (2) PTSD symptomatology plays a causal role in the development of guilt; (3) guilt and PTSD symptomatology are both products of a traumatic event, occurring alongside rather than causing one another; and (4) concepts closely related to guilt such as shame mediate the trauma-PTSD link and this process overlaps with guilt. Due to a lack of longitudinal and experimental research, supporting evidence for the respective models is scarce and causation and directionality of the guilt-PTSD relationship remain to be investigated (Pugh et al., 2015).

The present study aims to address this gap in empirical findings by experimentally assessing the first of the four putative models, which describes trauma-related guilt as part of a causal psychological mechanism driving the development of PTSD symptomatology. We thereby hope to clarify basic underlying principles of the guilt–PTSD relationship in order to advance the understanding of emotional factors influencing the development of the disorder.

Tilghman-Osborne, Cole, and Felton (2010) define guilt as involving ‘moral transgressions (real or imagined) in which people believe that their action (or inaction) contributed to negative outcomes’ (p. 546). Guilt is seen as the self’s negative evaluation of specific behaviours and thereby differs from the related concept of shame, where the entire self is negatively evaluated in a more stable manner (Lewis, 1971). This article will focus on the concept of guilt as defined by Tilghman-Osborne et al. (2010). In relation to traumatic events, this definition fits within the clinical model of guilt-based PTSD proposed by Lee et al. (2001). This model states that evaluation of the personal meaning of a traumatic event by the individual may crucially influence the development of PTSD. Four cognitive determinants of guilt related to personal involvement in traumatic events are frequently identified in traumatized patients (Kubany & Manke, 1995): (a) violation of personal standards of right and wrong; (b) perceived responsibility and preventability of the event; (c) perceived lack of justification for actions taken; and (d) false believes about pre-outcome knowledge and hindsight bias. Hereby, feelings of guilt are closely related to pre-trauma schemas patients have of themselves and others. Trauma-related information is matched to these schematic representations and both congruence and incongruence between trauma-related information and self-schemas can form the basis of intrusive activity as observed in PTSD patients. In the case of schema congruence, a traumatic event can lead to the activation or confirmation of underlying, guilt-associated beliefs about the self. These schemas then become the dominant mode of reasoning for the individual, causing him or her to understand the traumatic event from the perspective of the maladaptive schema. Guilt-charged intrusive recollections of the event resulting from this are linked to pervasive feelings of guilt and the occurrence of avoidance behaviours and rumination. In the case of schema incongruence, the traumatic event does not fit with underlying beliefs about the self. Beliefs about the self are not altered and therefore feelings of guilt are limited to the event-related memory (circumscribed guilt). Still, a feeling of violation of personal standards or responsibility may cause individuals to experience guilt-based intrusions that are characterized by replaying what happened in order to find indications when and how the individual could have acted differently to prevent the traumatic event (ruminative replay; Lee et al., 2001).

The putative model that is the subject of investigation in the current study fits within this clinical framework of guilt-based PTSD by describing guilt as part of the causal mechanism that drives the development of the disorder (Pugh et al., 2015). According to the model, a traumatic event can cause individuals to experience severe feelings of guilt, the degree of which depends on the perceived personal involvement. The evaluation of personal involvement may be influenced by factors such as perceived wrongdoing, responsibility, and self-blame (Foa & Rothbaum, 1999; Kubany et al., 1996). Feelings of guilt, in turn, may form the basis of trauma-related intrusions typical of PTSD symptomatology (Lee et al., 2001). Thus, this model proposes guilt to function as a meditational process underlying the development of PTSD following a traumatic experience.

As previously discussed, cross-sectional designs are not suited to determine causation and directionality of effects. In order to draw firmer conclusions about the nature of the guilt-PTSD link and to test whether the putative model presented by Pugh et al. (2015) is appropriate, experimental research can be used. Experimentally manipulating aversive states like guilt causes ethical issues, thus a non-clinical analogue population can be suitable for investigating basic psychological principles underlying the guilt-PTSD relationship. In addition to providing theoretical insights into non-anxiety factors that influence the development of PTSD, such research can have important implications for clinical practice, since enhanced understanding of the emotional profile underlying the disorder can help tailor interventions specifically to the needs of patients suffering from guilt-based PTSD (Dalgleish & Power, 2004; Power & Fyvie, 2012; Stapleton, Taylor, & Asmundson, 2006).

Even though research indicates a relationship between the emotion of guilt and the development of PTSD, the direction of this link remains to be established. The current study investigates, in an experimental setting, whether short-term feelings of guilt, induced as a reaction to an analogue trauma or stressor, can lead to PTSD-like phenomena such as intrusive thoughts at a non-clinical level. We thereby hypothesize that participants who are personally blamed for causing a computer crash and a related loss of data show an increased occurrence of intrusions and higher associated distress on the day of the incident, compared to a control group that is not personally blamed.

## 1. Method

### 1.1. Participants

A total of 51 first-year students (24 male, 27 female) from a Dutch university’s psychology programme participated in the study in exchange for course credit. The results of 11 participants were later excluded because they became suspicious of the deception that was used during the study. One participant was not taken into account for analyses since he did not complete the study due to high levels of distress experienced in reaction to the manipulation.

### 1.2. Measures

The Positive and Negative Affect Schedule (PANAS; Watson, Clark, & Tellegen, 1988) is a 20-item self-report measure to rate positive and negative affect experienced in the present moment. Each item is rated on a 5-point scale ranging from 1 (*very slightly* or *not at all*) to 5 (*extremely*) and the measure includes a guilt-item. Cronbach’s alpha was .88 in the current sample.

Intrusions and their characteristics were measured using an intrusion diary. The diary consists of questions to assess the number (‘How often did anything about the participation pop up spontaneously in your mind?’) and quality (‘Specify pop-up as thought, image, or feeling and give a short description of its content’) of intrusions experienced after participation, as well as the distress (‘How much were you bothered by the pop-up?’) caused by them. Distress of the pop-ups is rated on a 10-point scale ranging from 1 (*not bothered at all*) to 10 (*extremely bothered*).

The Trauma-Related Guilt Inventory (TRGI; Kubany et al., 1996) is a 32-item self-report measure assessing guilt experienced in relation to a specific traumatic event. Items are scored on a 5-point scale ranging from 4 (*extremely true*) to 0 (*not at all true*) and the measure includes three scales: (a) a four-item Global Guilt scale, measuring the magnitude of guilt experienced after a traumatic event; (b) a six-item Distress scale, measuring physical distress specifically related to the trauma memory; and (c) a 22-item Guilt Cognitions scale, measuring participants’ beliefs that their thoughts, feelings, or actions have violated personal and/or moral standards of behaviour. Cronbach’s alpha was .80 in the current sample.

The Perseverative Thinking Questionnaire (PTQ; Ehring et al., 2011) is a 15-item self-report measure assessing participants’ general tendency to engage in repetitive negative thinking as a reaction to negative experiences or problems. Items are scored on a 5-point scale ranging from 0 (*never*) to 4 (*almost always*). Cronbach’s alpha was .94 in the current sample.

Eysenck Personality Questionnaire – Revised (EPQ-R; Eysenck, Eysenck, & Barrett, 1985) is a self-report personality measure to assess the personality dimensions of Psychoticism, Extraversion, and Neuroticism. For this study, participants completed the Neuroticism scale (22 yes/no items), which places participants along the emotionally stable–emotionally unstable continuum based on the number of items they mark with yes. Cronbach’s alpha was .79 in the current sample.

The Brief COPE (Carver, 1997) is a 28-item self-report measure to assess strategies adopted to cope with stressful events in general. The questionnaire includes a self-blame subscale, consisting of two items that are scored on a 4-point scale ranging from 1 (*I usually don’t do this at all*) to 4 (*I usually do this a lot*). Cronbach’s alpha was .75 in the current sample.

### 1.3. Procedure

The experimental design included exposure to a computer crash and the subsequent (acted) distress of the researcher related to the loss of data. The design included two conditions: (1) blame (experimental condition), participants were personally blamed for causing the computer crash and the related loss of data; (2) no-blame (control condition), participants were explicitly told that the computer crash and the related loss of data were caused by technical failure and therefore not their fault. Participants were randomly assigned to one of the two conditions upon arrival in the laboratory. Thus, there was one between-subjects factor (blame vs. no-blame) as the independent variable. Reported number of intrusions and associated distress experienced by the participants were the dependent variables, which were assessed with the diary.

After arriving in the laboratory, participants were provided with an information sheet about the study and gave written consent for their participation. Participants were told that the study consisted of two parts, which were to be completed during two appointments, taking place on two consecutive days. Both parts belonged to the cover story, whereby participants were made to believe that Part 1 assessed changes in attitudes towards sustainable behaviour and Part 2 investigated the influence of different keyboard setups on typing performance. Participants completed a set of pre-manipulation measures, including the EPQ-R (Neuroticism), Brief COPE, PTQ, and PANAS. To increase credibility of the cover story, participants also completed a brief questionnaire on typing abilities. In addition, participants were instructed to complete the diary at home before going to bed on the day of the first appointment. Next, participants watched a brief video about a sustainability campaign after which Part 1 of the study was finished.

For Part 2, participants were introduced to the typing task. The computer task was based on a study by Horselenberg, Merckelback, and Josephs (2003), who used a similar setup to investigate the influence of false incriminating evidence on participants’ willingness to make false confessions. E-Prime 2.0 Professional was used to create the computer task. Stimuli appeared in lower case, black letters against a white background. Participants were informed that the task was designed to investigate the influence of different keyboard setups on typing speed and accuracy. All participants were told that they were assigned to the control group and, therefore, had to complete the task on a regular keyboard. The task consisted of typing five-letter combinations that appeared on the screen. It was stressed that participants should focus on typing as quickly and accurately as possible since their response time was recorded but that they should not press the SHIFT-key, because pressing that key may cause the computer to crash and data to be lost. To make the possibility of an inadvertent pressing of the SHIFT-key more plausible, the letters Q, A, and Z (all located close to the shift key) were presented in an increased frequency during the task. A total of 96 stimuli were presented before the computer crashed.

After the computer crash, the researcher either personally blamed the participants for pressing the shift key and thus causing the crash and the related loss of data (blame group), or told the participants that the crash was caused by technical failure and not the participants’ fault (no-blame group). Both groups experienced the stressor, which can be seen as an analogue trauma, of witnessing the researcher’s despair and helplessness following the incident and, except for holding the blame group responsible for causing the crash, both groups were treated identically by the researcher. To make participants in the blame group believe that they pressed the shift key, false incriminating evidence was presented (see Horselenberg et al., 2003) by telling participants that their responses, including pressing the shift key on trial 96, were visible on the researcher’s own computer screen. To evoke feelings of guilt in the blame group while avoiding defensive reactions by participants, emphasis was put on the researcher’s devastation in the face of the crash and loss of data. Participants were then asked to complete the PANAS for a second time (post-manipulation measure) after which the first appointment was finished. Before leaving the laboratory, participants were reminded to complete the diary before going to bed and were asked not to discuss the incident with their colleagues in order to keep future participants from learning about the deception.

Upon returning for the second appointment a day later, participants were asked to complete the follow-up measures, consisting of a third PANAS and the TRGI. After participants finished completing the questionnaires, they were fully debriefed, which included explaining the deceptive nature of the study and reminding participants not to discuss the study with their colleagues. The study has been approved by the Ethical Committee Psychology of the University of Groningen.

## 2. Results

### 2.1. Pre-manipulation measures

Final analyses included 39 participants, with 19 participants in the blame group (10 male, nine female) and 20 participants in the no-blame group (eight male, 12 female). To test whether groups differed with regard to certain personality characteristics and their general tendencies of reacting to stressful events and problems, participants completed a set of pre-manipulation measures, including the EPQ-R (Neuroticism), Brief COPE, and PTQ. Independent *t*-tests were performed to compare mean scores for the measures between the blame group and the no-blame group. Results showed no significant differences between groups for any of these three pre-manipulation measures (see Table 1).

### 2.2. Manipulation check for guilt

**Figure 1.**
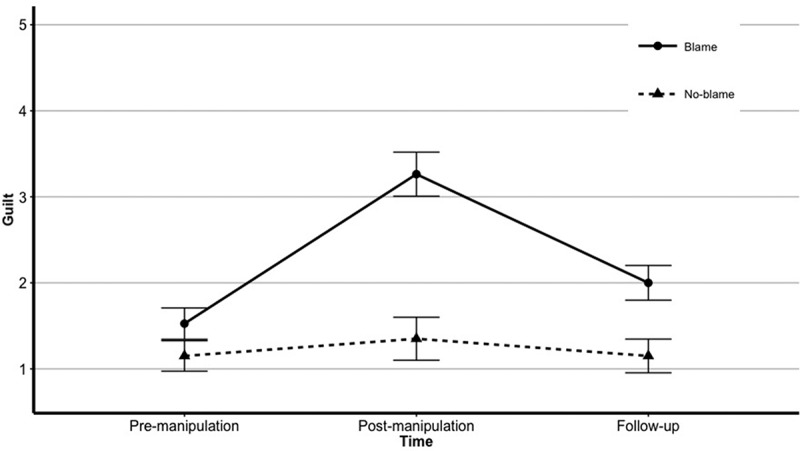
Changes in guilt-levels (assessed with the PANAS) over time for the blame group *n* = 19) and the no-blame group (*n* = 20). Error bars represent standard errors.

Figure 1 shows mean levels of guilt for both groups, as assessed with the PANAS guilt-item over time, for three points of measurement (pre-manipulation, post-manipulation, and follow-up). Since the aim of the current study was to investigate the influence of short-term induced guilt on intrusions, analyses were focused on pre-manipulation and post-manipulation guilt scores.

Purpose of the manipulation was to induce feelings of guilt in the blame group, so to test whether the manipulation was successful, a 2 (Group; blame, no-blame) x 2 (Time; pre-manipulation, post-manipulation) repeated measures ANOVA was performed. Results showed a significant main effect of Group, *F*(1,37) = 23.00, *p *< .001, *η^2^*= .38 and a significant main effect of Time, *F*(1,37) = 23.77, *p *< .001, *η^2^*= .39. Also, the Group * Time interaction was significant, *F*(1,37) = 14.09, *p *< .001, *η^2^*.28. Analyses of simple main effects revealed that there was no significant difference in guilt between the blame group (*M *= 1.52) and the no-blame group (*M = *1.15) for the pre-manipulation measure, *F*(1, 37) = 2.19, *p *= .147, *η^2^*= .05. For the post-manipulation measure, there was a significant difference in guilt between the blame group (*M *= 3.26) and the no-blame group (*M *= 1.35), *F*(1, 37) = 28.54, *p *= < .001, *η^2^*= .43. Further, analyses of simple main effects indicated that the increase in guilt between pre-manipulation and post-manipulation measures was significant for the blame group, *F*(1, 37) = 37.28, *p *= < .001, *η^2^*= .50, whereas the no-blame group did not show a significant increase in feelings of guilt between pre-manipulation and post-manipulation measures, *F*(1, 37) = 0.52, *p *= .475, *η^2^*= .01. Thus, the manipulation successfully induced feelings of guilt in the blame group. Besides measuring feelings of guilt, the PANAS also includes items regarding feelings of shame, distress, and feeling upset. Analyses of these emotions over time showed similar patterns as those observed for guilt, but changes in these feelings were less strong.

### 2.3. Intrusion frequency and related distress

**Figure 2.**
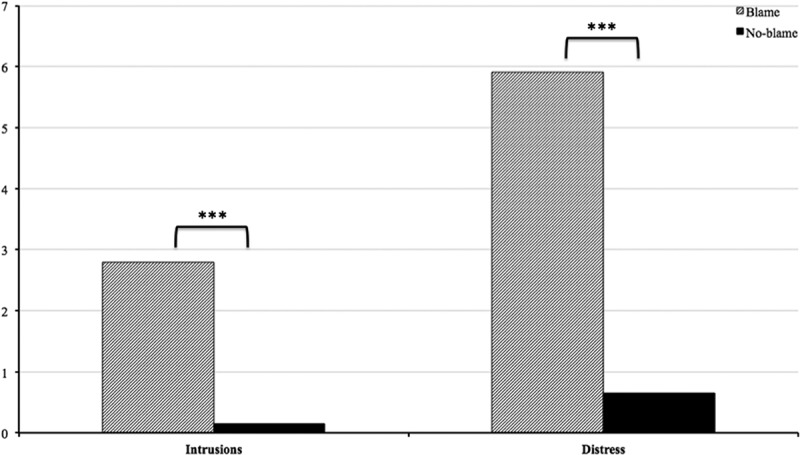
Mean number of intrusions and associated distress on the day of the incident (assessed with the diary) for the blame group (*n* = 19) and the no-blame group (*n* = 20). *** *p *< .001.

It was hypothesized that participants in the blame group would show an increased occurrence of intrusions and higher associated distress after the computer crash compared to participants in the no-blame group. To examine the link between the incident and intrusions, only incident-related intrusions were included for analyses. Selection of incident-related intrusions was based on descriptions of their content obtained from the diary. Independent *t*-tests were performed to compare the mean number of intrusions and associated distress between the two groups. Results showed that the blame group experienced significantly more intrusions (*M* = 2.79, *SD *= 2.55) than the no-blame group (*M* = 0.15, *SD *= 0.49), *t*(19.26) = -4.54, *p *< .001. Furthermore, the associated distress of the intrusions was significantly higher in the blame group (*M *= 5.91, *SD *= 3.36) compared to the no-blame group (*M *= 0.65, *SD *= 2.06), *t*(29.55) = −5.85, *p *< .001. Results therefore support the hypothesis that the blame group would experience a higher number of intrusions and associated distress compared to the no-blame group on the day of the incident (see Figure 2).

In order to test for the effect of guilt-levels on intrusion frequency and related distress, two univariate ANOVAs with post-manipulation levels of guilt as independent variable and number of incident-related intrusions as well as associated distress as dependent variables were performed. Results showed a significant effect of guilt-levels on both, incident-related intrusions *F*(1, 37) = 5.36, *p* = .002, *η^2^*= .38 and associated distress *F*(1, 37) = 9.63, *p* = < .001, *η^2^*= .53. Since the PANAS items ashamed, distressed, and upset also showed increased scores following the manipulation, ANOVAs with these emotions as independent variables were performed in order to test for a possible effect on number of incident-related intrusions and associated distress. Results indicate that none of these emotions had a significant effect on the intrusion frequency of the participants. Shame- and distress-levels did have a significant effect on the distress associated with the intrusions, but this effect was less strong than that of guilt-levels (*η^2^*= .28 and *η^2^*= .48 respectively).

To further explore whether the observed effect was indeed driven by guilt and not other emotions, shame and distress were independently added as covariate to the univariate analysis. Results showed that both shame and distress were no significant covariates and inclusion of these concepts did not change the effect of guilt on intrusion frequency or distress. This indicates that other negative emotions elicited by the stressor did not account for the effect on intrusions.

### 2.4. Additional measure of guilt

To allow for a multidimensional assessment of guilt in addition to the one-item guilt measure included in the PANAS, the TRGI was used to assess participants’ guilt-levels on the day following the computer crash (follow-up measure). Independent *t-*tests were performed to compare scores for the three TRGI scales (Global Guilt scale, Distress scale, and Guilt Cognitions scale) between the two groups and results showed that the blame group scored significantly higher on all three scales, indicating that they felt significantly more guilty and distressed in relation to the computer crash, compared to the no-blame group (see Table 1). Moreover, all three TRGI scale scores correlated strongly with post-manipulation PANAS guilt-scores, with Pearson’s correlation coefficients ranging from *r *= .70 to *r *= .79 (all *p*s < .001).

**Table 1.** Comparison of group means for pre-manipulation and follow-up measures.

		Blame	No-blame			
Time	Measure	*M* (*SD*)	*M* (*SD*)	*df*	*t*	*p*
Pre-manipulation	EPQ-R (N)	9.00 (4.40)	9.05 (4.67)	37	0.03	.973
	Brief COPE	5.60 (1.35)	5.50 (1.54)	37	−0.17	.866
	PTQ	3.21 (0.86)	2.90 (0.59)	37	−1.45	.156
Follow- up	TRGI-GG	1.90 (1.06)	0.59 (0.64)	29.33	−4.67	< .001
	TRGI-GC	1.55 (0.75)	0.68 (0.33)	24.60	−4.65	< .001
	TRGI-D	1.53 (0.96)	0.32 (0.39)	23.51	−5.14	< .001

GG = Global Guilt scale; GC = Guilt Cognition Scale; D = Distress scale.

## 3. Discussion

The present research aimed to gain insight in the role of guilt in the development of PTSD by experimentally assessing the influence of stressor-related guilt on the occurrence of stressor-related intrusions. Participants experienced a stressor that served as an analogue for trauma in the form a computer crash and related loss of data and were either personally blamed for causing the incident or were told that it was technical failure and therefore not their fault. We hypothesized that participants who showed elevated feelings of guilt after being personally blamed for causing the computer crash would experience an increased number of intrusions and higher associated distress on the day of the incident, compared to the group that was not personally blamed. Results showed that the number of intrusions was indeed significantly higher in the blame group. Moreover, the intrusions experienced in this group were rated as significantly more distressing compared to the intrusions experienced by the no-blame group. The increase in intrusion frequency and associated distress could not be explained by increases in other negative emotions (shame or distress) elicited by the stressor. Our findings therefore indicate that short-term induced guilt may contribute to the development of PTSD-like phenomena such as intrusive thoughts at a non-clinical level.

The staged computer crash was designed to provoke a strong perception of personal involvement by emphasizing participants’ wrongdoing, responsibility, and preventability in relation to the incident. As stated above, these factors may crucially influence the severity of experienced guilt, which can form the basis of intrusions typical of PTSD. Significantly higher TRGI Guilt Cognitions scale scores for the blame group indicate that this goal was achieved, since this scale assesses perceived violations of personal and/or moral standards of behaviour, including responsibility and preventability of the event. Regarding the changes of guilt-levels over time as assessed with the PANAS guilt-item, it is noteworthy that the level of guilt decreased significantly in the blame group between post-manipulation measure and follow-up measure, which took place on the day following the incident. The decrease in guilt-levels for this group could be explained by acknowledging that the stressor, which participants experienced in the form of the computer crash, was not strong enough to form the basis for prolonged feelings of guilt. Since the study aimed to investigate the effects of short-term induced guilt in a sample of healthy participants, the short-lived nature of heightened guilt-levels is preferable.

Besides the theoretical implications, clarifying the directionality of the guilt-PTSD relationship can help to improve the treatment of PTSD patients. Even though research points towards a diverse emotional profile underlying PTSD development, the alleviation of fear remains the main focus of therapeutic interventions (e.g. Shalev, Bonne, & Eth, 1996). Advancement in the understanding of emotional factors that underlie PTSD emphasizes possible benefits of more idiosyncratic treatment approaches that aim to change guilt-related feelings and cognitions associated with the traumatic event. Such interventions may be especially helpful for trauma groups that experience high personal involvement and therefore greater levels of guilt and more severe PTSD symptomatology (e.g. soldiers; Litz et al., 2009). In this context it needs to be noted that findings supporting the notion of increased trauma-related guilt intensifying PTSD symptomatology do not indicate, in turn, that a decrease in trauma-related guilt will help alleviate these symptoms. Further research is necessary to examine the effects of interventions targeted at guilt-related feelings and cognitions.

A few limitations should be taken into account for the present study. First, a non-clinical sample faced a stressor that served as an analogue for trauma. Analogue studies conducted in the laboratory, by definition, include stressors rather than actual trauma in order to investigate basic psychological principles that may influence the development of PTSD. Ethical considerations restrict the manipulation of aversive states, so naturally participants will never experience events that are considered traumatic according to DSM-5 criteria. Still, analogue methods like the trauma-film paradigm, which involves watching a film including traumatic events, have proved useful to study processes involved in trauma (James et al., 2016). Nevertheless, a critical stance towards the generalizability of laboratory findings to clinical practice is of great importance and such studies should aim to match real-life circumstances and the phenomena under scrutiny as closely as possible. Here, it can be debated whether a stressor counts as an analogue of trauma or not. The stressor participants faced in the present study was designed to provoke a strong perception of personal involvement by emphasizing participants’ wrongdoing, responsibility, and preventability of the event in order to make them feel guilty. Such personal involvement in a traumatic event may crucially influence the development of PTSD. We therefore think that the computer crash and related loss of data which participants experienced served as a suitable analogue for trauma. Even though the analogue nature of the present study needs to be considered when interpreting the results, this novel procedure can help to investigate psychological mechanisms underlying PTSD.

Second, we used a one-item measure to assess present-moment guilt in order to test our manipulation, asking: ‘How guilty do you feel right now?’ Tilghman-Osborne et al. (2010) criticize such short measures that leave the definition of guilt to the participant for not clearly delineating guilt from other related concepts. In our study, a brief and unobtrusive guilt measure that would not cause suspicion in the participants was necessary, therefore, the PANAS represented a suitable option. In addition, a multidimensional assessment of guilt in relation to the incident was conducted with the TRGI and strong correlations with the PANAS guilt-item affirm its suitability to assess guilt.

Third, the researcher was not blind for the condition, which might have strengthened the results in the hypothesized direction. These effects on the results might be considered minimal however, as the act of the researcher being in despair and feeling helplessness followed a standard protocol and interaction with the participant was avoided as much as possible to prevent any individual differences in stressor exposure.

Finally, one issue that complicates a clarification of the relationship between guilt and PTSD is a lack of consensus about how guilt relates to and distinguishes from the theoretical concept of shame (Kubany & Watson, 2003). Our manipulation aimed to induce guilt as defined by Tilghman-Osborne et al. (2010) by letting participants in the blame group believe that their action (pressing the SHIFT-key) led to a negative outcome (computer crash and loss of data). Results indicated that shame showed a similar pattern to guilt over time as assessed with the PANAS, however, guilt-levels showed a larger increase than shame-levels and scores of the TRGI scales confirm that substantial feelings of guilt in relation the to the computer crash were present in the blame group. Also, analyses of the effect of both guilt and shame on intrusion frequency and related distress showed a stronger effect of guilt that remained significant after controlling for shame. Thus, shame cannot explain the increase of number and distress of intrusions observed in the blame group.

The results of the present study provide valuable conceptual information about the relationship between guilt and PTSD. By assessing the influence of stressor-related guilt on the occurrence of intrusions in an experimental study, we found supportive evidence for the notion that feelings of guilt may foster PTSD-like phenomena and may therefore be part of a causal mechanism that drives the development of the disorder. Traditionally, PTSD is understood as an anxiety-based disorder. Clinical practise however shows that patients with PTSD experience an array of other negative emotions besides anxiety, including shame, anger, and guilt. The findings of this study show that incident-related guilt predicted increased subsequent PTSD symptomatology, supporting the potential importance of focussing on feelings of guilt in the treatment of PTSD.
